# New Insights Into Advanced Glycation End Products Induced Melanogenesis and Intervention Strategies

**DOI:** 10.1111/jocd.70521

**Published:** 2025-10-21

**Authors:** Xi Yang, Mengqi You, Huanjun Zhou, Zhen Li, Guangwen He, Thomas Mammone, Nadine Pernodet, Jian Cao

**Affiliations:** ^1^ The Estée Lauder Companies Innovation Shanghai China; ^2^ Clinique Laboratories Melville New York USA; ^3^ The Estée Lauder Research Laboratories Melville New York USA

**Keywords:** dimethoxytolyl propylresorcinol, glycation, green tea extract, hyperpigmentation, inflammation, melanogenesis, niacinamide

## Abstract

**Background:**

Skin hyperpigmentation refers to areas of skin that become darker than the surrounding skin due to an increase in melanin production, which greatly impacts skin esthetics. UV exposure, intrinsic hormonal changes, or injury are the general causes of hyperpigmentation. Recently, studies indicated that advanced glycation end products (AGEs) contribute to hyperpigmentation. However, detailed mechanisms involved in this process as well as therapeutic solutions are yet to be explored.

**Aims:**

This study aimed to investigate the underlying mechanisms as well as intervention strategies targeting AGEs‐induced melanogenesis.

**Methods:**

The study exposed skin cells and 3D epidermal models to AGEs and measured melanin production and tyrosinase activity. Dimethoxytolyl propylresorcinol (DP), niacinamide, and green tea extract were tested for their ability to inhibit AGEs‐induced melanogenesis or related cytokines. The AGEs breaking efficacy of DP was assessed on glycated lysozyme by mass spectrometry, as well as on glycated skin cells and 3D full‐thickness skin models.

**Results and Conclusions:**

Exposure to AGEs stimulates tyrosinase activity and melanin production in melanocytes and 3D melanocyte‐containing epidermal skin models. AGEs upregulate IL‐33 expression in fibroblasts via NLRP1/Caspase1 activation. DP was discovered to be an effective AGEs crosslink breaker and also capable of inhibiting AGEs induced melanin production in skin models. Moreover, niacinamide and green tea extract effectively inhibit IL‐18/IL‐33 secretion stimulated by AGEs. Those 3 components work collectively to target different stages of AGEs induced melanogenesis in skin and appear to be a potent intervention strategy to treat skin hyperpigmentation and sallowness issues during the aging process.

**Abbreviations**
AGEs: advanced glycation end products
DP: dimethoxytolyl propylresorcinol
RAGE: the receptor for advanced glycation end products

## 1 Introduction

Melanocytes, pigment‐producing cells of follicular and interfollicular epidermis, generate melanosomes and melanin [1]. The quantity of melanin produced and transferred to keratinocytes, followed by its incorporation, aggregation, and degradation, influences the overall complexion and coloration of the skin. Changes in melanin production or distribution can result in a skin disorder called hyperpigmentation. Hyperpigmentation is a broad term that includes various skin discolorations, pigmentation, and darkening‐related disorders, such as melasma, post‐inflammatory hyperpigmentation, and lentigines [2]. Those changes can result from a range of internal and external factors, including hormonal fluctuations, inflammation, injury, acne, eczema, medication, UV exposure, and more.

Glycation is a naturally occurring, nonenzymatic Maillard reaction between free reducing sugars and proteins, DNA, and lipids. The reaction begins early in life, develops clinical symptoms around the age of 30, and progressively accumulates in tissues and skin due to the glycated proteins that are difficult to decompose [3]. During the process, a variety of complicated glycation products produced at different stages and pathways were collectively referred to as advanced glycation end products (AGEs). AGEs contribute to cellular dysfunction by modifying intracellular molecules and accumulate in tissues with aging [4, 5]. In skin, AGEs accumulate over time and are exacerbated by exogenous factors, for example, ultraviolet radiation, resulting in wrinkles, impaired elasticity, and other skin issues. Exogenous AGEs consumption and endogenous AGEs production activate numerous signaling pathways through a series of receptors [6]. The type I receptor for AGEs (RAGE) from the immunoglobulin (Ig) superfamily is the most extensively studied receptor in humans. Other AGEs receptors, including the AGE‐R1, ‐R2, and ‐R3 receptors, as well as scavenger receptors such as stabilin‐1 and stabilin‐2, have also been identified [7]. Previously, it has been demonstrated that accumulation of AGEs in both epidermis and dermis affects skin tone and leads to yellow discoloration of the skin, as AGEs by themselves are of brownish color. Recently, AGEs have been shown to also impact skin color by promoting melanogenesis through RAGE, indicating that AGEs could be a new contributor to skin hyperpigmentation [8], while the detailed mechanism remains to be illustrated.

The inflammasomes are innate immune system receptors and sensors that regulate the activation of caspase‐1 [9]. In the previous study, it was concluded that excessive AGEs promote melanin production by activating the NLRP3 inflammasome and enhancing IL‐18 secretion in fibroblasts [10]. Based on the previous understandings of inflammasomes, NLRP1 and NLRP3 inflammasomes were both stimulated in irradiated human skin fibroblasts, while NLRP1 but not NLRP3 promotes senescence and the senescence‐associated secretory phenotype (SASP), indicating a key role of NLRP1 as a stress sensor in human skin fibroblasts [11]. Indeed, compared with NLRP3, NLRP1 is highly expressed in keratinocytes and is considered the most predominant inflammasome sensor in human skin [12], which activates caspase‐1 to cleave and activate cytokines including IL‐1β and IL‐18 [6, 13]. In addition to IL‐18, many other inflammatory factors secreted by fibroblasts have also been shown to increase melanogenesis, including IL‐33, PGE2, and PGF2 [7]. Therefore, the investigation of the holistic roles of inflammasome/inflammatory signaling in different skin cells under an AGEs‐rich environment is still needed.

Additionally, as hyperpigmentation is a common cosmetic concern, effective treatment options are required to address AGEs‐induced pigmentation. The formation of AGEs was deemed an irreversible chemical process, and to attenuate the adverse impact of AGEs on skin, most strategies focused on inhibiting the formation of AGEs through the protection of the amino groups of substrate proteins, carbonyl group scavenging, metal cation chelation, or ROS neutralization [14]. However, a group of deglycation agents, also known as AGEs‐breakers, was also identified, such as ALT‐711 (Alagebrium) and phenylacetyl thiazolium bromide (PTB) [15], providing a new potential approach for the reversal of AGEs‐induced pigmentation.

In the present study, the melanogenesis pathway induced by AGEs was explored in melanocytes and a reconstructed melanocyte‐containing epidermis model. We also studied AGEs‐promoted inflammatory factors in keratinocytes and fibroblasts, respectively. The upstream signaling pathways involving NLRP1, ASC, and caspase‐1 were investigated. For the first time, we discovered dimethoxytolyl propylresorcinol (DP), previously known as a potent tyrosinase inhibitor, is also capable of degrading AGEs with the possibility of preventing AGEs' adverse impact on skin cells. This article addresses a cosmetic treatment strategy with a combination of DP, niacinamide, and green tea extract to intervene in AGEs induced hyperpigmentation at multiple steps.

## 2 Materials and Methods

### 2.1 Preparation of AGEs

AGEs were generated by glycating BSA or bovine skin collagen as previously described with slight modification [16, 17, 18]. BSA (A1933, Sigma) was dissolved in PBS, and bovine skin collagen I (Biocell, Xi'an, China) was dissolved in 0.017 N acetic acid. The solutions were incubated with methylglyoxal (M0252, Sigma) respectively at 37°C for 5 days; afterwards, 30 mL of the solutions were put in a Slide‐A‐Lyzer Dialysis Cassette (87 737, Thermo Scientific) and extensively dialyzed against 2 L PBS or 0.017 N acetic acid as per the manufacturer's instructions for 48 h. The dialysis buffer was changed every 4 h.

### 2.2 Cell Culture

Human dermal fibroblasts (HDFs) from female donors were obtained from Biocell (Xi'an, China). Cells were recovered and maintained in Dulbecco's modified Eagle medium (DMEM) (Corning, NY, USA), supplemented with 10% fetal bovine serum (FBS) (Corning) and 1% penicillin–streptomycin (Corning), in an atmosphere of 5% CO_2_ at 37°C.

Normal human epidermal keratinocytes (NHEKs) and normal human epidermal melanocytes (NHEMs) were purchased from Biocell (Xi'an, China). Cells were recovered and maintained in specific medium from Biocell, respectively, supplemented with growth factors and glutamine, in an atmosphere of 5% CO_2_ at 37°C.

### 2.3 Immunofluorescence Staining of Cells

HDFs/HEKs were seeded in 24‐well plates at a density of 2 × 10^4^ cells per well and treated after 24 h of adherence. Niacinamide (purchased from DSM under the trade name NIACINAMIDE PC/B3 FRESH) and green tea extract (purchased from IKEDA under the trade name GYOKURO EXTRACT) were solubilized in water and diluted to the working solutions in culture medium. Cells were treated w/o AGEs for 48 h or treated with niacinamide/green tea extract for 24 h and washed with PBS prior to incubation with AGEs for 48 h. The concentrations used are at a nontoxic level.

For immunofluorescence staining, as previously described [10], the cells were then fixed with 4% paraformaldehyde (PFA), permeabilized with 0.1% Triton X‐100, and blocked with PBS containing 1% BSA and 10% goat serum for 1 h. Cells were further incubated with primary antibody overnight at 4°C with slow rocking, followed by incubating with secondary antibody at 1:1000 dilution for 1 h at RT. Finally, cells were counterstained with DAPI and imaged with Operetta CLS High Content Imaging System.

The primary antibodies were anti‐CML (1:200, #ab125145, Abcam, Cambridge, UK), anti‐IL33 (1:200, #ab187060), anti‐NLRP1 (1:200, #ab36852), anti‐IL18 (1:200, PA5‐79479, Invitrogen, Waltham, MA), and anti‐ASC (1:300, sc‐514 414, Santa Cruz Biotechnology, Texas, USA). The secondary antibodies were Goat Anti‐Mouse IgG H&L (Alexa Fluor 594) (#ab150116) and Goat Anti‐Rabbit IgG H&L (Alexa Fluor 488) (#ab150077).

### 2.4 Melanin Content, Distribution, and Tyrosinase Activity Assay

To examine the effect of AGEs (glycated‐BSA) on melanogenesis, melanin content, distribution, and tyrosinase (TYR) activity were detected in NHEMs and reconstructed 3D melanocyte‐containing epidermal skin models (Melakutis, Biocell).

Dimethoxytolyl propylresorcinol (DP, CAS No.: 869743‐37‐3, purchased from UNIGEN under the trade name Nivitol) was solubilized in ethanol and diluted to the working solutions in culture medium to treat the NHEMs or applied topically on Melakutis. NHEMs were treated with AGEs at different concentrations or AGEs w/o DP for 48 h. Melakutis were incubated with AGEs added to the culture media, w/o DP or ethanol topically applied for 5 days.

To determine the melanin content [19], the cell pellet or skin model was dissolved in 1 mL of 1 N NaOH/10% DMSO for 1 h at 80°C. Then, 200 μL of solubilized melanin was added into 96‐well plates, and optical density values were measured at 405 nm and compared with a standard curve of synthetic melanin with a microplate reader, afterwards normalized by protein concentration. To measure the tyrosinase activity, melanocytes treated w/o AGEs were washed with ice‐cold PBS and lysed with 0.5% sodium deoxycholate (Sigma) in distilled water at 0°C for 1 h [20]. After quantification of the protein levels and adjustment of concentrations with lysis buffer, the lysate was incubated with 0.1% L‐dopa (Sigma) in 0.1 M PBS buffer (pH 6.8) at 37°C. Tyrosinase activity was assayed using L‐dopa as the substrate. Formation of dopachrome, which has an absorbance maximum at 475 nm, was spectrophotometrically determined at different time points between 0 and 60 min. To determine the melanin distribution, Fontana Masson staining was performed on cells fixed with 4% PFA or a 3D epidermal skin model processed with paraffin sections with Masson‐Fontana Kit (ml094682, Mlbio).

### 2.5 Enzyme‐Linked Immuno Sorbent Assay (ELISA) to Determine Inflammatory Factors

Levels of IL‐18 (#ab215539), IL‐33 (#ab223865), PGE2 (#ab316263), and PGF2 (#ab133056) were detected using ELISA kits according to the manufacturer's instructions. The absorbance at 450 nm was measured with a microplate reader and compared with the standard curve. The concentrations of IL‐18, IL‐33, PGE2, and PGF2 were normalized to that of the protein content of each sample.

### 2.6 SDS–PAGE, Western Blotting, and Caspase Activity Assay

Western blotting and sodium dodecyl sulfate–polyacrylamide gel electrophoresis (SDS–PAGE) were carried out in accordance with conventional procedures. Briefly, cells in the exponential growth phase were washed once with PBS; then, the cells were immediately lysed and boiled. Each sample was subjected to SDS–PAGE, followed by transfer to a PVDF membrane (Millipore) for western blot analysis. After the membranes were blocked for 1 h at room temperature in 5% (w/v) BSA in PBS containing 0.1% (v/v) Tween‐20 (PBST), they were probed with primary antibodies overnight at 4°C in antibody dilution buffer (QuickBlockTM, Beyotime). Following incubation with horseradish peroxidase (HRP)‐conjugated secondary antibodies in 5% BSA, the protein bands were developed using a Sensitive ECL Kit (Med Chem Express) and visualized using an iBright 1500 system (Invitrogen).

The primary antibodies were anti‐caspase 1 (1:1000, PA5‐38099, Thermo Fisher Scientific), anti‐NLRP1 (1:1000, #ab36852), anti‐RAGE (1:1000, #ab216329), anti‐ASC (1:5000, sc‐514 414), and anti‐GAPDH (1:10 000, 60004–1‐Ig, Proteintech). The secondary antibodies were anti‐mouse HRP‐conjugated (1:5000, RGAM001, Proteintech) and anti‐rabbit HRP‐conjugated (1:5000, RGAR001, Proteintech) recombinant antibodies.

Caspase activity was detected using the Caspase‐Glo 1 Inflammasome Assay Kit (G9951, Promega) according to the manufacturer's instructions. Briefly, 40 000–60 000 cells were seeded in a 96‐well plate and treated after 24 h of adherence. After 48 h of treatment, the 96‐well plate containing cells was removed from the incubator and equilibrated at RT for 5 min. Add 100 μL of Caspase‐Glo 1 Reagent and Caspase‐Glo 1 YVAD‐CHO Reagent respectively to the wells of no‐cell controls, treated cells, and vehicle controls. Then, gently mix the contents of the wells using a plate shaker at 300–500 rpm for 30 s. The plate was incubated at RT for at least 1 h to allow the luminescent signal to stabilize. The luminescence was measured with a plate‐reading luminometer [21].

### 2.7 In‐Tube Deglycation Analysis of Glycated Protein Using Mass Spectrometry

The method was adapted from previous literature [22] with minor modifications. Glycated lysozyme (gl‐lysozyme) was first prepared by incubating lysozyme with ribose. Lysozyme was initially dissolved in deionized water to a final concentration of 0.500% (w/w). Subsequently, a solution containing 0.400% (w/w) lysozyme and 0.500% (w/w) ribose in deionized water (pH 6) was prepared. The mixture was incubated for 168 h at 37°C and stopped via freeze‐drying.

A 1.000% (w/w) glycated lysozyme solution was prepared in deionized water, and a 5% (w/w) solution of the active compound in DMSO was prepared prior to use. The deglycation medium, with or without 0.1% (w/w) of the active compound, was prepared to achieve a final protein concentration of 0.5% (w/w) and 20% (w/w) in DMSO. The solutions were stored at 37°C. Samples were collected at three time points: the initial stage, after 1 day, and after 3 days.

Mass spectrometry analysis was performed to evaluate the deglycation efficiency. Samples were analyzed on a triple quadrupole mass spectrometer (TQS‐Micro, Waters). Source temperature and desolvation temperature were set to 80°C and 150°C, respectively. The collision energy, cone voltage, and capillary voltage were set to 10 eV, 35 V, and 2.5 kV, respectively. Mass spectra were acquired in positive ion mode over an m/z range of 800–1900. Sample solutions were introduced via infusion at a flow rate of 15 μL/min. Instrument control, data acquisition, and processing were performed using MassLynx v4.2 software.

The neutral molecular mass of lysozyme is 13 681.3 Da. In the mass spectrum of the blank sample, ion signals corresponding to [M + 11H]^11+^ (m/z≈1301), [M + 11H + ribose]^11+^ (m/z≈1313), [M + 10H]^10+^ (m/z≈1431), and [M + 10H + ribose]^10+^ (m/z≈1444) were observed. The glycation rate of lysozyme was determined by calculating the intensity ratio of m/z≈1301 to m/z≈1313.

### 2.8 Determine De‐Glycation Efficacy in Cellular & 3D Full‐Thickness Skin Model

NHEKs were seeded in 24‐well plates at a density of 2 × 10^4^ cells per well. After 24 h of adherence, cells were treated with fresh medium containing 400 μM methylglyoxal as a glycation inducer, or a control medium without methylglyoxal, for 48 h. Cells were then washed twice with DPBS, and fresh medium supplemented with or without DP was added for 24 h. Glycation was detected by immunofluorescence staining with anti‐CML antibody as described before.

Full thickness skin models (FulKutis, Biocell) were treated with fresh medium containing 5 mM methylglyoxal as a glycation inducer, or a control medium without methylglyoxal, for 96 h. Models were then washed with DPBS, and fresh medium was added. DP was added topically on the surface of the models for another 72 h. The medium and treatment were refreshed every day. Afterwards, the models were washed in PBS, L* & b* values were recorded by Spectrometer DSM II ColorMeter, and the auto‐fluorescence at wavelengths of ex 370 nm/em 440 nm, which is ascribed to detecting AGEs, was measured in a microplate reader. For CML staining, the skin models were embedded in OCT before cryosectioning to 5 μm slides, and CML was detected with anti‐Carboxymethyl Lysine antibody [CML26] (1:200, ab125145, Abcam). The fluorescent images were captured using a Leica DM IL LED, and the fluorescence intensities were calculated by Image‐Pro Plus Image processing software [23, 24].

### 2.9 Statistical Analysis

We used Prism 6.0 software (GraphPad Software) to perform the statistical analysis. All statistical analyses comparing two groups were performed with the unpaired Student's *t* test.

## 3 Results

### 3.1 AGEs Promote Melanogenesis

**FIGURE 1:**
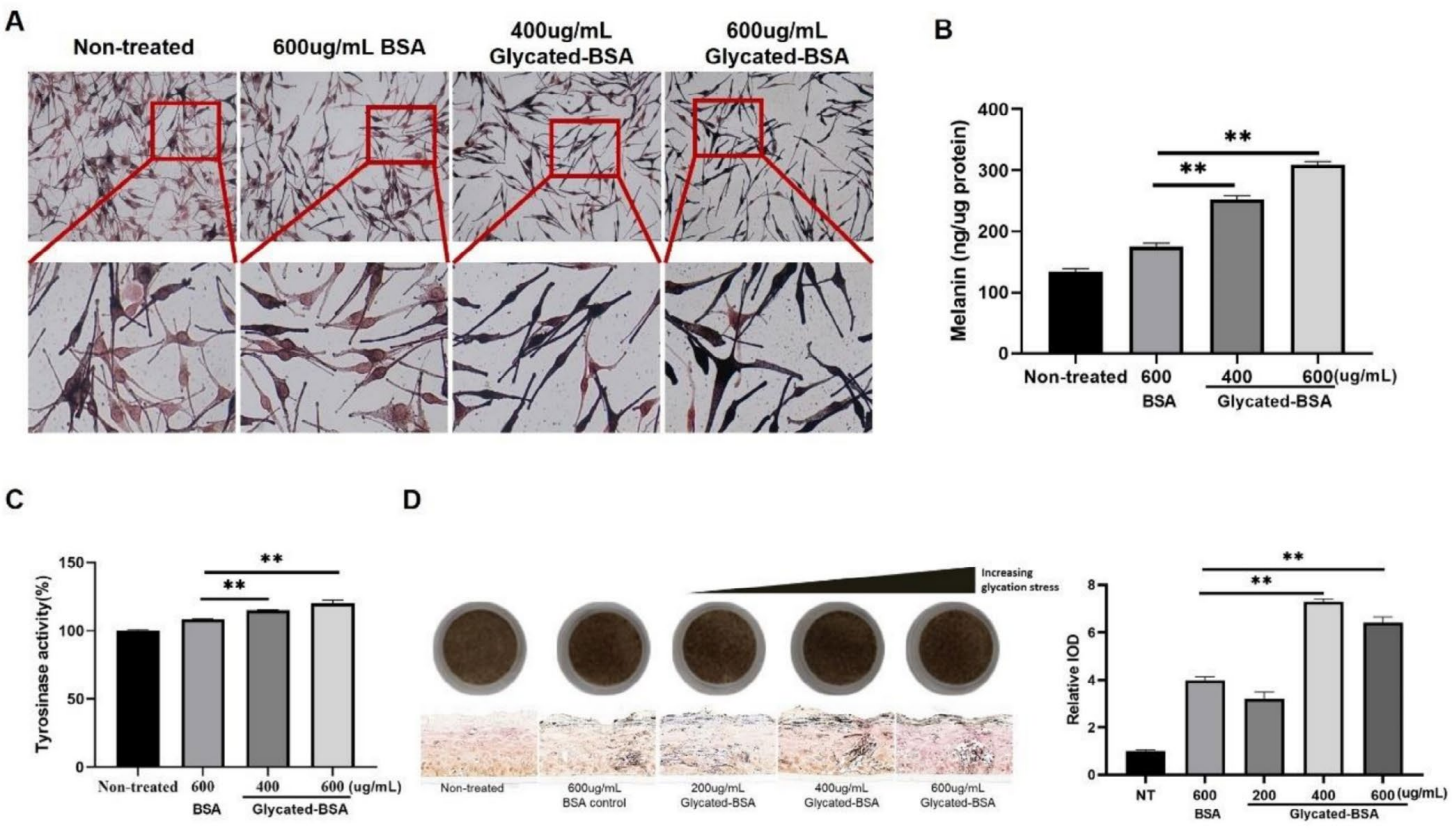
AGEs increase melanin production and tyrosinase activity. NHEMs (A–C) and Melakutis (D) were treated with BSA or glycated‐BSA of different concentrations, and melanin content (B), tyrosinase activity (C) and melanin distribution (A, D) were measured. Values are presented as mean ± SD. Statistical significance was assessed using an unpaired *t*‐test (**p < 0.01.)

To determine the effects of AGEs on melanogenesis in melanocytes, NHEMs and a 3D melanocyte‐containing epidermal model (Melakutis) were treated with different dosages of glycated‐BSA, melanin content was measured, and melanin distribution was shown by Fontana Masson staining. AGEs at various dosages were confirmed noncytotoxic prior to treatments (data not shown). Results indicated that AGEs could activate melanin production in melanocytes in a dose‐dependent manner (Figure 1A,B,D), by upregulating the activity of tyrosinase, which is the key enzyme involved in melanogenesis (Figure 1C). These findings collectively demonstrated that AGEs expedite melanogenesis through activating tyrosinase in melanocytes.

### 3.2 Stimulated Production of Inflammatory Factors by AGEs in NHEKs and HDFs

**FIGURE 2:**
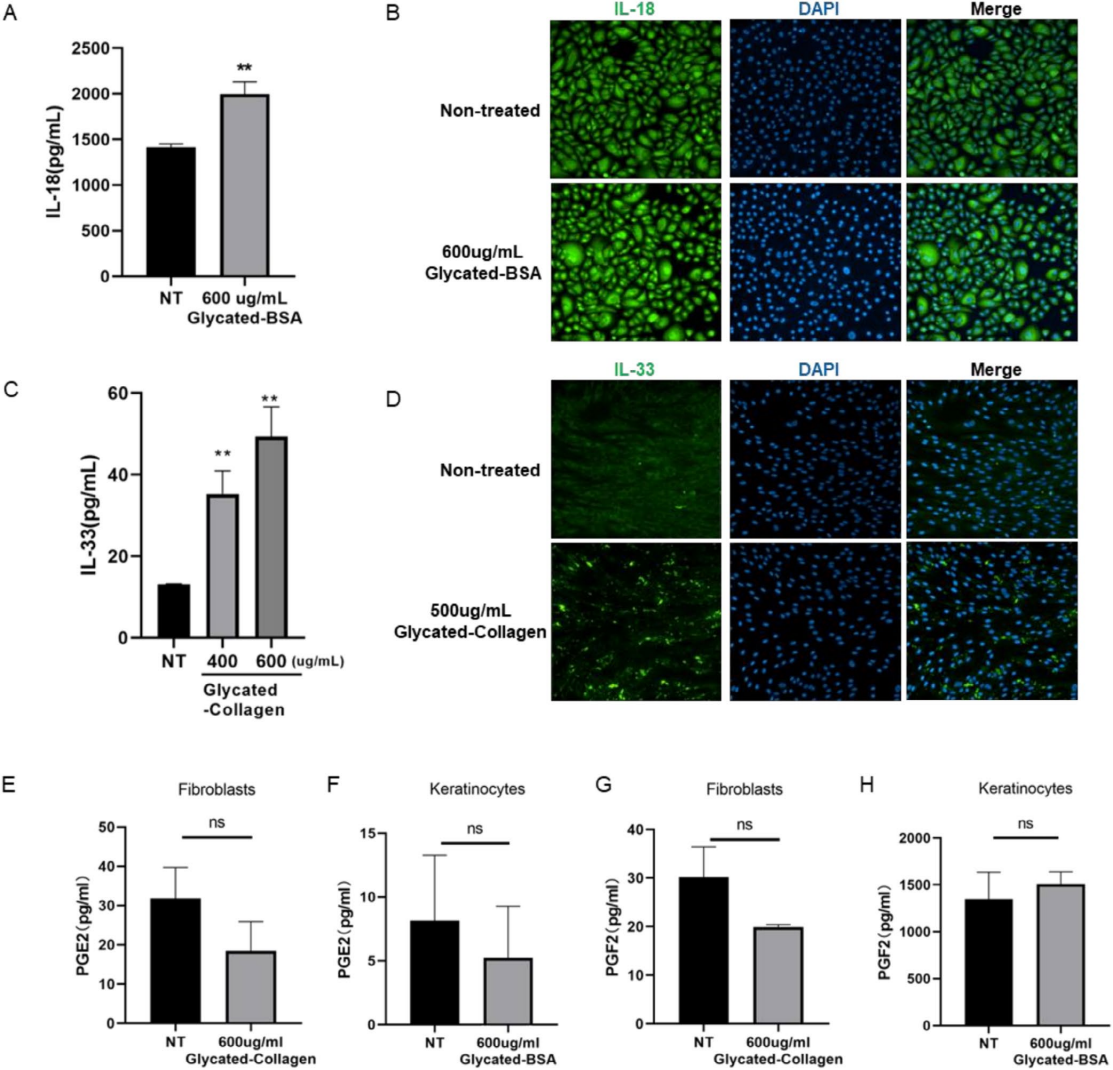
AGEs promote melanogenesis by enhancing the production of IL‐18 and IL‐33 in human skin cells. NHEKs (A, B, F, H) were treated with 600 μg/mL glycated‐BSA and HDFs (C, D, E, G) were treated with 600 μg/mL glycated‐collagen for 48 h, cellular expression of IL‐18 (A), IL‐33 (C), PGE2 (E, F) and PGF2 (G, H) were measured by ELISA and immunofluorescence staining (B, D) respectively. Values are presented as mean ± SD. Statistical significance was assessed using an unpaired *t*‐test (**p < 0.01.)

Several secreted factors have been reported as influencers of melanogenesis within melanocytes [7]. Keratinocytes secrete substances like IL‐18, IL‐33, GM‐CSF, PGE2, and PGF2, which promote melanogenesis in melanocytes. Similarly, fibroblasts release factors such as IL‐33, PGE2, and PGF2, which also stimulate this process. These inflammatory factors influence melanogenesis in melanocytes through paracrine signaling. To determine which factor(s) are involved in the regulation of melanin synthesis by AGEs, we extensively examined several factors that have been mentioned above. The results indicated that IL‐18 was significantly upregulated by AGEs in NHEKs (Figure 2A,B), and IL‐33 was significantly upregulated by AGEs in HDFs (Figure 2C,D), whereas PGE2 and PGF2 were not impacted evidently (Figure 2E–H).

### 3.3 AGEs Activate NLRP1 Inflammasome Through RAGE Signaling

**FIGURE 3:**
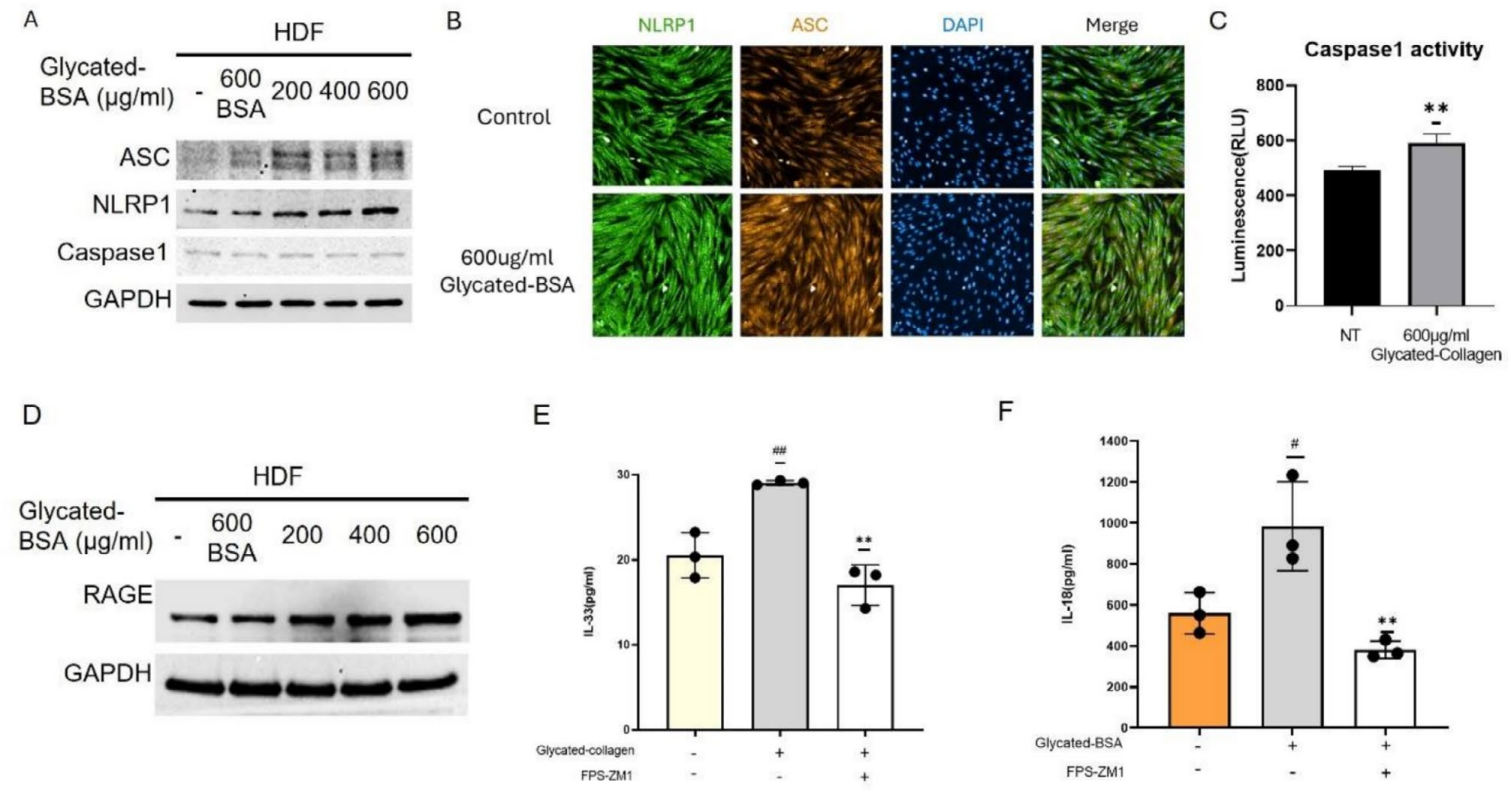
AGEs activate NLRP1 inflammasome through RAGE signaling. Cellular expression of NLRP1, ASC, Caspase‐1 and RAGE in HDFs stimulated by different dosages of BSA or glycated‐BSA were analyzed with Western Blot (A, D), and colocalization of ASC and NLRP1 (B) and caspase‐1 activity (C) in HDFs induced with 600 μg/mL glycated‐BSA/collagen for 48 h were also measured. Cellular expression of IL‐33 in HDFs was analyzed with ELISA, after incubation with/without 600 μg/mL glycated‐collagen/FPS‐ZM1 for 48 h (E), and expression of IL‐18 in NHEKs was analyzed with ELISA, after incubation with/without 600 μg/mL glycated‐BSA/FPS‐ZM1 for 48 h (F). Values are presented as mean ± SD. Statistical significance was assessed using an unpaired *t*‐test (^#^ *p* < 0.05; **, ^##^ *p* < 0.01.)

Glycated proteins can interact with various cellular receptors, such as the RAGE, leading to a cascade of signaling events that enhance melanin synthesis. Because AGEs have been found to affect the activation of NLRP1 and NLRP3 inflammasomes [25], we then analyzed the markers of activated NLRP1 inflammasome, a high expression complex in fibroblasts. As shown in Figure 3A and Figure 3B, protein levels and immunofluorescence intensity of NLRP1 and ASC were significantly increased in glycated‐BSA treated fibroblasts compared with those in the control group. An elevation in caspase‐1 activity instead of caspase‐1 expression level was also observed in fibroblasts treated with AGEs (Figure 3A,C).

Also, our findings indicate that glycated BSA increases the protein expression level of RAGE (Figure 3D), which is consistent with previous reports. Additionally, in the presence of the RAGE antagonist FPS‐ZM1, the expression of IL‐33 in HDFs and IL‐18 in NHEKs (Figure 3E,F) was restored, suggesting that AGEs elevated the expression of IL‐33 and IL‐18 through RAGE.

### 3.4 Cosmetic Therapeutic Approaches to Inhibit AGEs Induced Melanogenesis Stepwise

**FIGURE 4:**
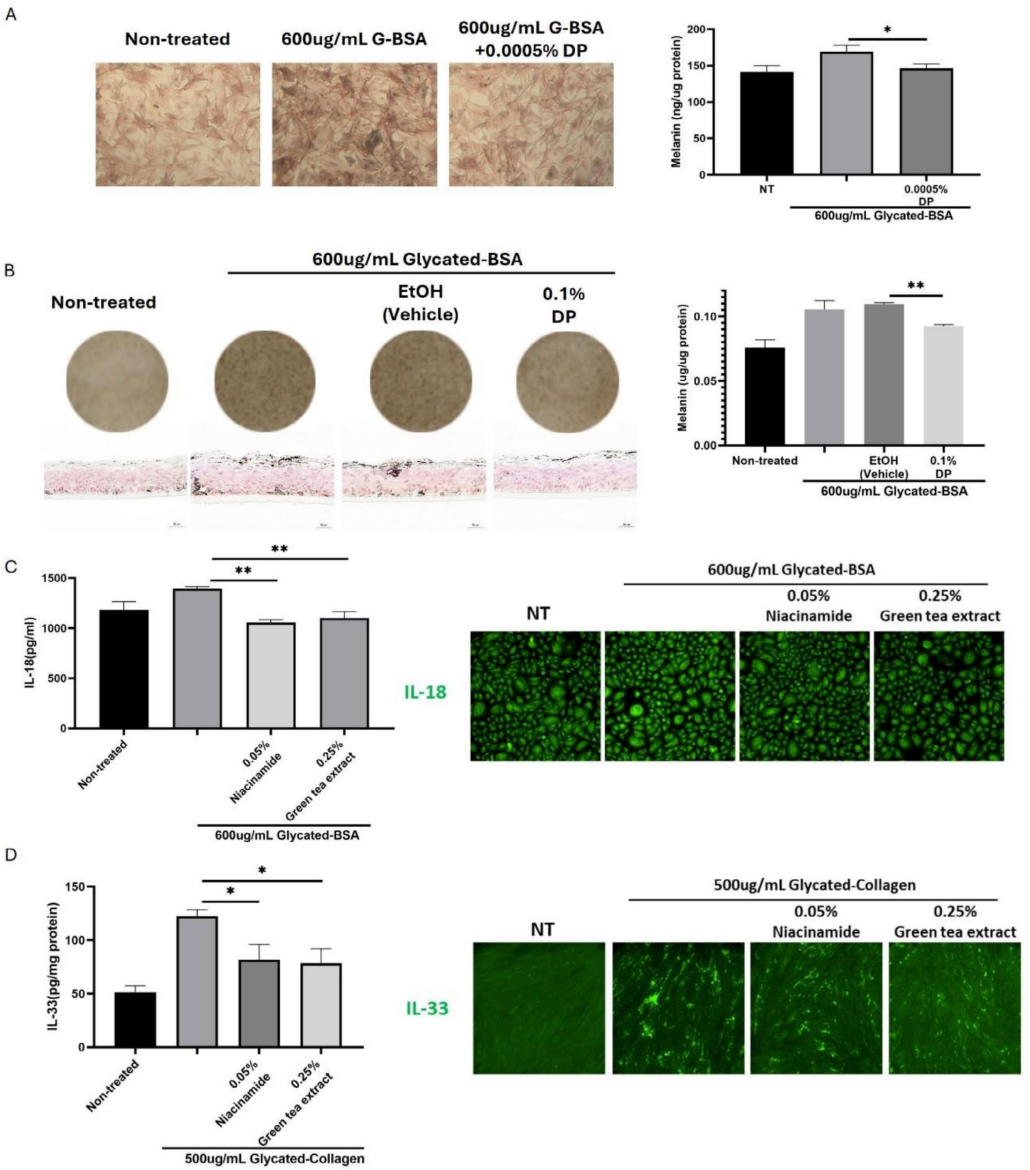
Cosmetic therapeutical complex to inhibit AGEs induced melanogenesis at multiple steps. DP inhibits the melanin production included by 600 μug/mL glycated‐BSA in NHEMs (A, Fontana Masson staining in left panel and melanin content in right panel) and Melakutis (B, visual pictures and Fontana Masson staining in left panel and melanin content in right panel). Cells were treated with Niacinamide or green tea extract 24 h prior to incubation with AGEs for 48 h, the secretion of IL‐18 (C, ELISA in left panel and immunofluorescence staining in right panel) in NHEKs and the secretion of IL‐33 (D, ELISA in left panel and immunofluorescence staining in right panel) in HDFs were measured. Values are presented as mean ± SD. Statistical significance was assessed using an unpaired *t*‐test (*p < 0.05; **p < 0.01.)

After revealing the mechanisms of AGEs induced melanogenesis, we then aim to target each step with cosmetic actives to achieve comprehensive management of pigmentation across the entire pathway. The actives are known to have skin brightening effects including resorcinol derivative DP, niacinamide, and green tea extract. Therefore, we first investigated the efficacy of these actives in inhibiting AGEs induced melanogenesis. Firstly, we tested the effect of DP on melanocytes. The results demonstrated that co‐treatment of DP with AGEs significantly inhibits AGEs induced melanin production in NHEMs (Figure 4A). Also, topical application of DP inhibits AGEs induced melanogenesis in the 3D epidermal model Melakutis (Figure 4B). Based on our previous laboratory studies, we subsequently investigated the effects of niacinamide and green tea extract on inflammatory factor secretion induced by AGEs. NHEKs and HDFs were treated with niacinamide or green tea extract 24 h prior to incubation with AGEs for 48 h. According to the data from ELISA and immunofluorescence, both niacinamide and green tea extract were able to inhibit the synthesis and secretion of IL‐18 from NHEKs (Figure 4C) and IL‐33 from HDFs (Figure 4D), indicating the inhibitory effect towards the upstream signaling pathway of melanin synthesis induced by AGEs.

### 3.5 Degradation of AGEs by DP

**FIGURE 5:**
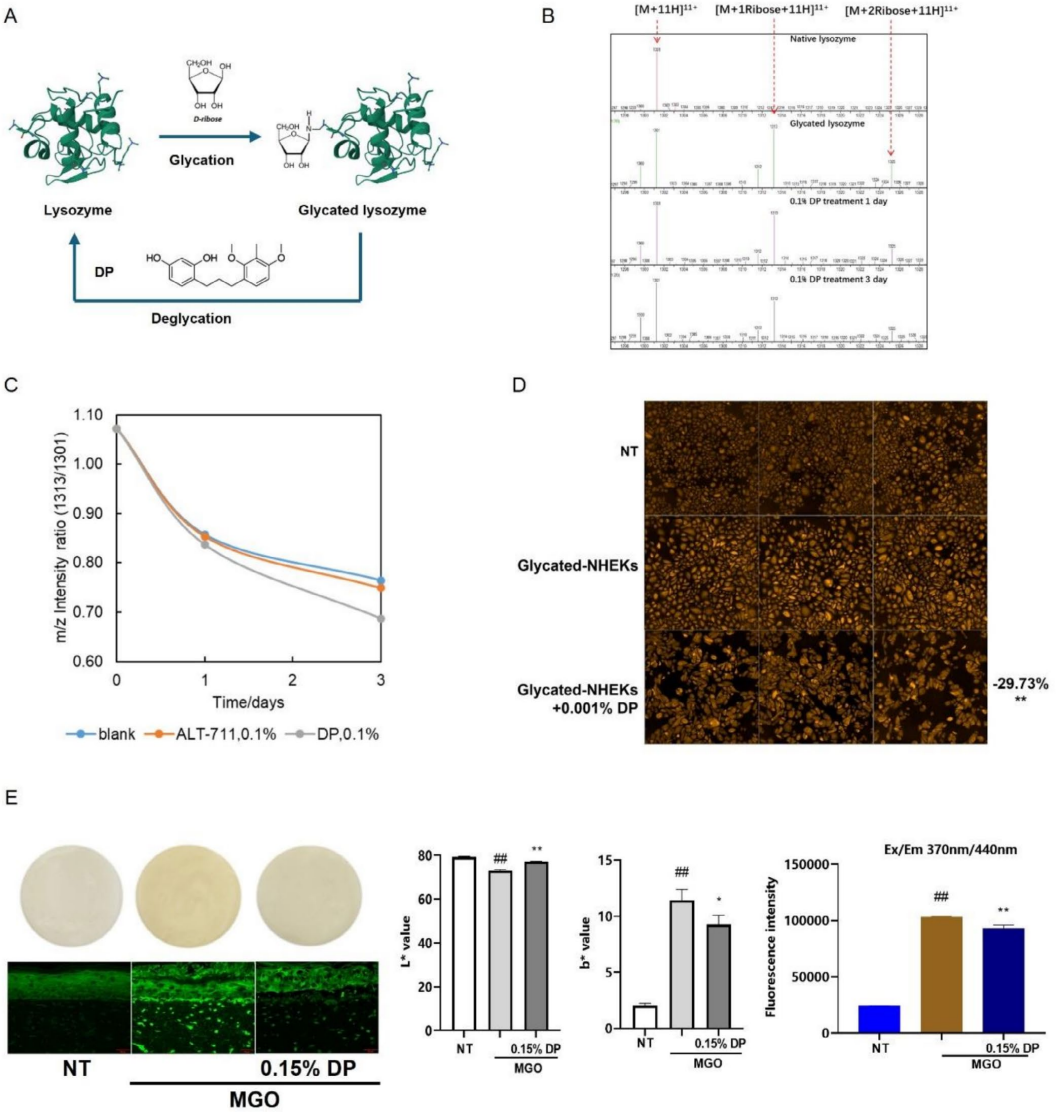
DP reverses the glycation process in different experimental models. Hypothetical mode of action of protein deglycation by DP (A). Mass spectrum verification of the protein glycation and deglycation process by determining the intensity ratio of m/z≈1301 to m/z≈1313 (B), and the effect of DP on R1313/1301 over time with ALT‐711 served as the positive control (C). CML immunofluorescence staining was performed on non‐glycated NHEKs, glycated NHEKs, and glycated NHEKs treated with 0.001% DP (D). Visual pictures (E, left upper panel), L* & b* values (E, middle panels), autofluorescence at Ex/Em 370 nm/440 nm representing AGEs signal (E, right panel), and CML immunofluorescence staining on cryosection slides (E, left lower panel) were measured for non‐treated Fulkutis, glycated Fulkutis, and glycated Fulkutis topically treated with 0.15% DP. Values are presented as mean ± SD. Statistical significance was assessed using an unpaired t‐test (^#^ *p* < 0.05; **, ^##^ *p* < 0.01.)

In addition to antagonizing the melanin production and melanogenesis factors simulated by AGEs, we also aim to target AGEs directly and explore AGEs degradation approaches. Though AGEs are generally considered as products of irreversible reactions, a group of chemicals was reported as AGE‐breakers, which could degrade AGEs by breaking the cross‐linking and have deglycation efficacy. Here we first investigate the role of DP in breaking crosslinks of AGEs in a test tube assay; lysozyme was employed as a model protein (Figure 5A). After glycation induction, the content of glycated lysozyme was determined in HPLC (Mass Spectrum). When it was incubated together with DP, the ratio of glycated protein in whole composition decreased evidently (lower than negative control as well as positive control, ALT‐711) (Figure 5B,C) indicating DP as a deglycating reagent by breaking AGEs cross‐linking. Furthermore, we investigate its efficacy on skin cells. In NHEKs glycated by MGO, after removal of glycation stress and application of 0.001% DP, the glycation level represented by CML staining was further reversed by 29.73% compared to non‐treated controls (Figure 5D). The efficiency had also been validated on full thickness skin models (Fulkutis) (Figure 5E). After 4 days of treatment with MGO to induce glycation, MGO residue was removed and skin models were washed and further topically treated with 0.15% DP for another 3 days. Autofluorescence measured at Ex/Em 370 nm/440 nm representing AGEs signals decreased ~10% with significance, which is consistent with CML immunostaining results (Figure 5E). Notably, the skin models treated with DP also showed significantly decreased darkness and yellowness measured by L* and b*, indicating that by degrading AGEs, DP could help to reverse the sallowness resulting from glycation.

## 4 Discussion

### 4.1 DP as a Potent Inhibitor of AGEs Induced Melanogenesis on Melanocyte and 3D Epidermal Skin Models via Inhibiting Tyrosinase Activity

Methylglyoxal (MGO) is a highly reactive molecule and has been widely established as an AGEs inducer [26]. The brownish end products of glycation known as AGEs have been known to contribute to skin yellowness, especially in the process of photoaging. Recently, AGEs have also been shown to impact hyperpigmentation, such as dark spots and uneven skin tone, by promoting melanin overproduction. In the present study, we firstly confirmed that AGEs can expedite melanocyte activity by enhancing tyrosinase activity, and tested the inhibition efficacy of DP (CAS#869742‐37‐3), originally isolated from the medical plant *Dianella ensifolia* and then synthesized synthetically, which had been reported to be not only an effective tyrosinase inhibitor 22 times more potent than kojic acid but also effective in promoting tyrosinase degradation and is able to treat visible signs of hyperpigmentation clinically [27, 28]. In this study, DP is shown to significantly inhibit AGEs‐induced melanin overproduction, which is also suggested to be closely related to its strong tyrosinase inhibition ability.

### 4.2 Niacinamide/Green Tea Extract to Regulate AGEs Induced Secretive Melanogenesis Inflammatory Signals in NHEKs/HDFs

Excess melanogenesis and irregular melanin deposition are more severely promoted by the inflammatory process in acne lesions [7, 29]. Interleukin‐18 (IL‐18) can activate melanocytes by binding to its specific receptor, IL‐18R, which is expressed on the surface of these cells [30]. This interaction triggers intracellular signaling cascades that enhance the activity of melanocytes. In addition, interleukin‐33 (IL‐33) is a cytokine that has emerged as a significant player in the regulation of melanogenesis. IL‐33 binds to its specific receptor, ST2 (also known as IL1RL1), which is expressed on the surface of melanocytes [31]. This interaction triggers the activation of intracellular signaling pathways, including the nuclear factor‐kappa B (NF‐κB) and mitogen‐activated protein kinase (MAPK) pathways [32]. Previous reports have shown that AGEs promote the secretion of IL‐18 in HDFs through NLRP3 inflammasome activation. In this study, we also investigated other inflammatory factors and inflammasomes involved in the AGEs‐stimulated pathways and in various skin cell types. The results show that in addition to HDFs, AGEs can also stimulate IL‐18 secretion in NHEKs. Our data is consistent with the previous studies that caspase‐1 catalyzes the maturation of pro‐IL18 and pro‐IL33 [6]. Moreover, in HDFs, the secretion of IL‐33 is also increased, possibly through NLRP1 inflammasome activation. These findings suggest the assembly and activation of the inflammasome induced by AGEs treatment. As the increase could be suppressed by FPS‐ZM1, a RAGE inhibitor, we concluded that AGEs can be recognized by RAGE in NHEKs and HDFs and stimulate the secretion of IL‐18 and IL‐33 through NLRP1 inflammasome activation. We further investigated the inhibitory effect of several known soothing compounds, including niacinamide and green tea extract. The results show both niacinamide and green tea extract as potent inhibitors of IL‐18/IL‐33 secretion under AGEs stress.

### 4.3 DP Promotes AGEs Degradation in Acellular and Skin Cells/Models

In addition to the attenuation of the melanin overproduction signal induced by AGEs, we also investigated the possibility of directly degrading AGEs to reduce the original stress. The cross‐linking of protein with AGEs through ‐SH/‐NH2 groups is generally deemed to be irreversible [33] and results in tissue stiffness and critical fracture lengths changed by limiting collagen fibril intermolecular sliding [34], which is known to be involved in the role of the development of renal, ophthalmologic, neurological, and cardiovascular complications in diabetes and the aging cycle [35]. Thus, breaking AGEs cross‐linking could also be a potential therapeutic approach to those aging diseases. Several novel molecules, such as ALT‐711 and PTB (phenyl thiazolium bromide) as AGEs breakers, were studied previously [33, 36]. We here studied the potential efficacy of DP in degrading AGEs crosslinks using ALT‐711 as a positive control. To our surprise, DP is demonstrated to break down AGEs effectively from in‐tube assays, cellular models, as well as a 3D full‐thickness skin model. Notably, the treatment of glycated skin models with DP results in a significant reversal of AGEs signal and sallowness, indicating that DP can not only treat visible signs of hyperpigmentation by tyrosinase inhibition but also contribute to the reversal of sallowness by degrading AGEs. And thus, the protein structure is potentially renewed and re‐functionalized in the skin. The detailed mechanism of degradation of AGE crosslinks is still not yet elucidated. Hypothetically, strong antioxidant capacity and structural uniqueness are possible reasons [28]. Further studies are required to understand at the molecular level.

### 4.4 Holistic Cosmetic Treatment Strategy to Block AGEs Induced Melanogenesis in Skin

**FIGURE 6:**
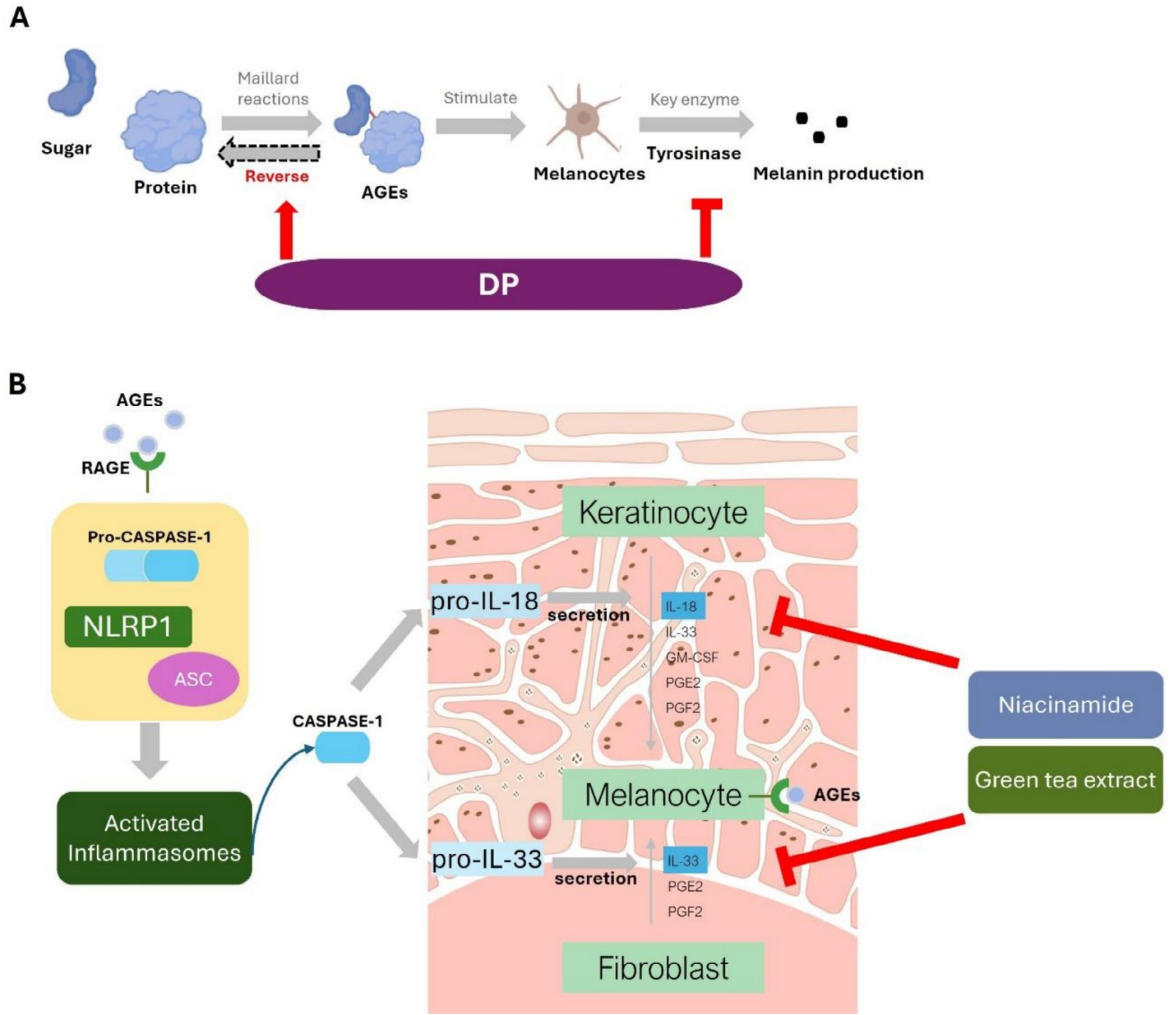
Schematic illustration of multi‐steps intervention mechanism of cosmetic therapeutic complex comprising DP/niacinamide/green tea extract in intervention of AGE induced melanogenesis: (A) DP works to break AGE crosslinks between sugar and protein, as well as decrease melanin overproduction by inhibiting tyrosinase activity induced by AGEs in melanocytes. (B) AGEs activate downstream inflammatory signals via binding to RAGE, promote Caspase‐1 activity to secrete IL‐18 and IL‐33 in NHEKs and HDFs, respectively. Niacinamide and green tea extract can effectively inhibit those inflammatory factors from release and thus inhibit following melanocyte overactivity.

Combined with the findings discovered in this study that AGEs are involved in the whole cycle of melanogenesis by impacting not only melanocytes but also inducing inflammation signals from paracrine cells (NHEKs and HDFs), we hereby built a comprehensive cosmetic strategy to help counter these effects for a healthier skin appearance at different cellular levels. As described in Figure 6, it comprises:

1. DP to break the crosslinks of AGEs.
2. DP to inhibit tyrosinase activity and melanin overproduction in melanocytes exposed with AGEs.
3. Niacinamide/green tea extract to inhibit IL‐18 production in NHEKs, IL‐33 expression in HDFs, respectively.

## 5 Conclusion

In this investigation, we have demonstrated the mechanism of glycation on hyperpigmentation based on 3D skin models, cells, and in tube experiments. We have discovered that the accumulation of AGEs stimulates melanogenesis by enhancing the activity of tyrosinase, the rate‐limiting enzyme in the melanogenesis pathway. The inflammasome NLRP1 also plays a vital role in this process. AGEs upregulate the expression and co‐localization of NLRP1 and ASC, which promotes the activity of caspase‐1. This, in turn, elevates the secretion of IL‐18 in NHEKs and IL‐33 in HDFs, allowing crosstalk across all layers of the skin and eventually resulting in melanin buildup. Moreover, we found that AGEs elevated IL‐18 and IL‐33 secretion via RAGE.

Upon elucidating the aforementioned mechanisms, we then targeted each step and screened actives to achieve a more comprehensive inhibition of melanogenesis across the entire pathway. This includes the use of DP to inhibit melanin overproduction in melanocytes and 3D epidermal models induced by AGEs, as well as the use of niacinamide and green tea extract to suppress the production of the cytokines IL‐18 and IL‐33. Despite these advances, there is still an unmet need for compositions and procedures that can effectively reverse the damage caused by AGEs, particularly in the skin, where the accumulation of these end products contributes to age‐related deterioration. Notably, we discovered that DP can degrade AGEs, reverting them to ordinary/pristine proteins, thereby reducing the level of glycation and reducing the chronic damage that AGEs inflict on the skin.

## Author Contributions

**Xi Yang:** conceptualization and methodology; **Mengqi You:** methodology, validation, investigation, and original draft; **Huanjun Zhou:** conceptualization, review and editing, visualization; **Zhen Li:** methodology and investigation; **Guangwen He:** supervision; **Thomas Mammone:** review and editing; **Nadine Pernodet:** review and editing; **Jian Cao:** resources, project administration, funding acquisition.

## Consent

The authors have nothing to report.

## Conflicts of Interest

The authors are employees of Estée Lauder Companies and thus declare no conflicts of interest.

## Data Availability

The data that support the findings of this study are available on request from the corresponding author. The data are not publicly available due to privacy or ethical restriction.
